# Nonfocal transient neurological attacks are related to cognitive impairment in patients with heart failure

**DOI:** 10.1007/s00415-019-09376-z

**Published:** 2019-05-21

**Authors:** Eline A. Oudeman, Jacoba P. Greving, Astrid M. Hooghiemstra, Hans-Peter Brunner- La Rocca, Geert Jan Biessels, L. Jaap Kappelle, Heart-Brain Connection Consortium, M. J. Daemen, M. A. van Buchem, R. J. van der Geest, M.J.P. J. P. van Osch, A. de Roos, W. M. van der Flier, A. C. van Rossum, H. P. Brunner La Rocca, M. A. Ikram, P. J. Koudstaal, W. J. Niessen, R. van Oostenbrugge, M. L. Bots, G. J. Biessels, L. J. Kappelle

**Affiliations:** 1 Department of Neurology and Neurosurgery, Brain Centre Rudolf Magnus, University Medical Centre Utrecht, Utrecht University, Utrecht, The Netherlands; 2 Department of Neurology, OLVG West, Amsterdam, The Netherlands; 3 Julius Centre for Health Sciences and Primary Care, University Medical Centre Utrecht, Utrecht University, Utrecht, The Netherlands; 4 Department of Neurology, Alzheimer Center Amsterdam, Amsterdam Neuroscience, Vrije Universiteit Amsterdam, Amsterdam UMC, Amsterdam, The Netherlands; 5 Department of Medical Humanities, Amsterdam Public Health Research Institute, Amsterdam UMC, Vrije Universiteit Amsterdam, Amsterdam, The Netherlands; 6 Department of Cardiology, Maastricht University Medical Center, Maastricht, The Netherlands

**Keywords:** Cognitive functioning, Cognitive impairment, Transient neurological attacks, Heart failure

## Abstract

**Introduction:**

Nonfocal transient neurological attacks (TNAs) are associated with an increased risk of future dementia, but it is unclear whether TNAs are also associated with concurrent cognitive impairment. We hypothesized that recent TNAs are related to worse cognitive functioning. We tested our hypothesis in patients with heart failure, as these patients are at risk of cerebral hypoperfusion, which might play a role in the etiology of TNAs.

**Methods:**

We performed neuropsychological testing in all patients with heart failure enrolled in the Heart Brain Connection study. We assessed global cognition, attention-psychomotor speed, executive functioning, memory and language. All patients were interviewed with a standardized questionnaire on the occurrence of TNAs in the preceding 6 months. We studied associations between TNAs and cognitive functioning with linear and logistic regression analyses, adjusted for age, sex and education. We performed additional analyses in patients without previous stroke or TIA and in patients without brain infarction on MRI.

**Results:**

Thirty-seven (23%) of 158 patients (mean age 70 years, 67% men) experienced one or more TNAs. Patients with a recent TNA were more likely to be impaired on ≥ 1 cognitive domains than patients without TNAs [41% vs. 18%, adjusted odds ratio 4.6, 95% confidence interval (CI) 1.8–11.8]. Patients with TNAs performed worse than patients without TNAs on global cognition (mean difference in *z* scores − 0.36, 95% CI − 0.54 to − 0.18), and on the cognitive domains attention-psychomotor speed (mean difference − 0.40, 95% CI − 0.66 to − 0.14), memory (mean difference − 0.57, 95% CI − 0.98 to − 0.15) and language (mean difference − 0.47, 95% CI − 0.79 to − 0.16). These associations were independent of cardiac output and volume of white matter hyperintensities. Subgroup analyses in patients without previous stroke or TIA or brain infarction on MRI (*n* = 78) yielded comparable results, with the exception of the cognitive domain language, which was no longer different between patients with and without TNAs.

**Conclusion:**

Among patients with heart failure, TNAs are associated with cognitive impairment, which warrants the need for more clinical awareness of this problem.

**Electronic supplementary material:**

The online version of this article (10.1007/s00415-019-09376-z) contains supplementary material, which is available to authorized users.

## Introduction

Nonfocal transient neurological attacks (TNAs) are characterized by an acute onset of neurological deficits, such as unsteadiness, confusion or bilateral weakness [1, 2]. In contrast to transient ischemic attacks (TIAs), the signs and symptoms of TNAs cannot be attributed to one specific arterial territory of the brain [3]. TNAs are associated with an increased risk of stroke, coronary artery disease and dementia [2, 4, 5]. Given this increased risk of future dementia, TNAs might be associated with concurrent cognitive impairment, despite the fact that signs and symptoms of TNAs are by definition transient. The relation between TNAs and concurrent cognitive impairment, however, has never been investigated.

The etiology of TNAs is unknown, but cerebral hypoperfusion, with or without ischemic damage of the brain, has been described as an important causal factor [1, 5]. Patients with heart failure are at risk of cerebral hypoperfusion, which is thought to be mediated through a reduced cardiac output, and related to the NYHA classification and duration of heart failure [6–8]. Besides cerebral hypoperfusion, these patients are also known to be at increased risk of cognitive impairment [9, 10]. We, therefore, investigated the relation between TNAs and cognitive functioning in patients with heart failure. We hypothesized that, in these patients, a recent TNA is related to worse cognitive functioning through a compromised cardiac output. We performed additional analyses in patients without a history of stroke or TIA, and in patients without brain infarction on magnetic resonance imaging (MRI), as these factors may independently influence cognitive functioning.

## Methods

### Study population and design

Between September 2015 and August 2018, patients with heart failure were recruited from four cardiology outpatient clinics in the Netherlands [11]. The inclusion criterion was an established diagnosis of heart failure that had been clinically stable for at least 6 months. Heart failure was defined according to the European Cardiology Society Guidelines as both signs and symptoms typical of heart failure with objective evidence of a structural or functional abnormality of the heart at rest on routine echocardiography [12]. All patients were independent in daily living and were able to undergo cognitive testing and magnetic resonance imaging (MRI) of the brain. Those with a history of dementia or with a Mini Mental State Examination (MMSE) < 24 were excluded. Other exclusion criteria were: a life expectancy less than 3 years unrelated to heart failure, atrial fibrillation at the time of inclusion, premature ventricular contractions exceeding 10% of the total number of heart beats, and a neurological or psychiatric diagnosis affecting cognitive functioning (e.g. Parkinson’s disease or substance abuse).

The current study is embedded in the Heart Brain Connection (HBC) study, a multicenter cohort study that focusses on the cardiovascular and hemodynamic contributions to cognitive impairment. Detailed information on the rationale and design has been described elsewhere [11]. The HBC study was approved by the ethics committee at the Leiden University Medical Center. All participants provided written informed consent.

### Classification of TNAs

All participants were interviewed by a trained physician or research nurse with a standardized questionnaire on the occurrence of eight nonfocal symptoms in the preceding 6 months (Table 1). TNAs were defined as attacks with one or more nonfocal signs or symptoms with an acute onset, a minimum duration of 30 s and a maximum duration of 24 h. Symptoms that were compatible with a different diagnosis, such as migraine or epilepsy, were excluded from the analysis. The interviewer was blinded to the neuropsychological test scores.

**Table 1:** Predefined nonfocal neurological symptoms

Unconsciousness
Confusion
Amnesia
Unsteadiness
Bilateral leg weakness
Blurred vision
Nonrotatory dizziness
Paresthesias

All symptoms should have an acute onset, a minimum duration of 30 s and a maximum duration of 24 h

### Neuropsychological assessment

Cognitive functioning was assessed in detail with an extensive and standardized neuropsychological test battery that was based on the Dutch Parelsnoer Initiative [13]. The MMSE [14] was used as a screening instrument. Memory was assessed with the Rey Auditory Verbal Learning Test (RAVLT) [15] total immediate recall, RAVLT delayed recall, RAVLT recognition and the Visual Association Test (VAT) [16] (short version). Language was assessed with the VAT (naming) and the 1-min animal fluency [17, 18]. The Trail Making Test (TMT) [19] part A, Stroop Color Word Test (SCWT) [20–22] I and II, Letter Digit Substitution Test [23] and Digit span [24] forward were used to assess attention and psychomotor speed. Executive functioning was assessed with the TMT B/A index, SCWT interference and Digit span backward. Global cognitive functioning was calculated by combining the compound *z* scores of all four cognitive domains. Patients’ performances on all individual neuropsychological tests were compared with a reference group that consisted of 128 people that were recruited among spouses and relatives of patients [11]. The reference group was free from heart failure, and the same exclusion criteria as for heart failure patients were applied.

### Brain imaging

MRI of the brain was performed on 3T scanners (Philips Ingenia, Philips Achieva and Philips Gemini; Philips Medical Systems, Best, the Netherlands). We acquired T1-, T2- weighted and flair-attenuated inversion recovery (FLAIR) sequences. A neuroradiologist who was blinded to clinical information rated all MRI scans on the presence of brain infarction, which was defined as either one or more cortical, subcortical or lacunar infarcts. Furthermore, white matter hyperintensities of presumed vascular origin were measured on FLAIR sequences in milliliters (mL).

### Clinical assessment

Cardiac output was assessed with MRI of the heart, left ventricular ejection fraction was assessed by echocardiography, and duration of heart failure was derived from medical records. We assessed age, sex, level of education, NYHA classification (divided in seven categories from class I to IV), smoking status, a history of myocardial infarction, peripheral arterial occlusive disease, TIA, ischemic stroke, hypertension and the presence of diabetes mellitus, chronic obstructive pulmonary disease (COPD), obstructive sleep apnea syndrome (OSAS) and the use of antihypertensive or lipid-lowering medication by means of a standardized interview. Systolic and diastolic blood pressures were measured during the study visit on the left and right arm with an automatic oscillometric BP monitor; the mean of these two readings was used for analyses.

### Statistical analysis

Descriptive analyses characterize the study population of heart failure patients with and without TNAs. Differences were determined with independent sample *t* tests, Chi square tests or Fisher exact tests when appropriate.

All neuropsychological test scores were standardized into individual *z* scores using the mean and standard deviation (SD) of control participants of the HBC study as a reference group. Individual *z* scores of each test were subsequently combined into cognitive domains. Global cognitive functioning was calculated as the mean *z* score of all cognitive domains. We considered a cognitive domain as impaired when the *z* score was below − 1.5 of the reference group, which was further dichotomized into impairment of ≥ 1 and ≥ 2 cognitive domains.

We used linear regression analyses with the presence of TNAs as independent variable and *z* scores of each cognitive domain as well as global cognition as dependent variable (separate analyses for each cognitive domain and global cognition). We used logistic regression analysis to study the association between TNAs and cognitive impairment (separate analyses for impairment of ≥ 1 and ≥ 2 cognitive domains). In both analyses, we performed three different adjustments. First, we adjusted for age, sex and education. Second, we additionally adjusted for volume of white matter hyperintensities on MRI (in milliliters, mL). Third, additional adjustments were made for cardiac output.

Subgroup analyses were performed after excluding patients with a history of stroke or TIA or patients with brain infarction on MRI.

All analyses were done with IBM SPSS Statistics version 24.0 (IBM Corp, Armonk, NY).

## Results

A total of 162 patients with heart failure were included in the HBC study. After exclusion of three patients with missing questionnaires on nonfocal symptoms and one patient with missing neuropsychological test scores, 158 patients [mean age 69.7 years (SD 9.8), 67% men] remained for analyses. Characteristics of the study population are described in Table 2. Thirty-seven patients (23%) had experienced one or more TNAs in the preceding 6 months. Patients with TNAs more often had a history of TIA than patients without TNAs (*P* = 0.025). Patients with and without TNAs were comparable in age, sex, educational level and presence of hypertension. Furthermore, cardiac output, left ventricular ejection fraction, duration of heart failure and NYHA classification did not differ between patients with and without TNAs.

**Table 2:** Baseline characteristics

	No TNA (*n* = 121)	≥ 1 TNA (*n* = 37)	*P* value
Male	85 (70)	21 (57)	0.126
Age (years)	70.0 ± 10.0	68.5 ± 9.0	0.395
Education, median (Q1–Q3)	5 (4-6)	5 (4-6)	0.560
Smoking status			0.789
Current	17 (15)	5 (14)	
Former	68 (56)	23 (62)	
Never	36 (29)	9 (24)	
Hypertension	65 (54)	20 (56)	0.846
Antihypertensive medication	95 (83)	27 (75)	0.312
Lipid-lowering medication	73 (63)	22 (59)	0.705
Diabetes mellitus	22 (19)	3 (8)	0.138
COPD	13 (11)	4 (11)	0.946
OSAS	13 (11)	7 (19)	0.191
History			
Myocardial infarction	60 (50)	20 (54)	0.634
TIA	8 (7)	7 (19)	0.025
Ischemic stroke	6 (5)	3 (8)	0.469
PAOD	6 (5)	2 (5)	0.914
Blood pressure (mmHg)			
Systolic	134 ± 19	135 ± 15	0.856
Diastolic	76 ± 10	77 ± 13	0.769
LVEF (%)	43 ± 8	44 ± 10	0.616
Cardiac output (L/min)	5.3 ± 1.2	5.2 ± 1.2	0.375
NYHA classification			0.655
Class I	56 (46)	12 (32)	
Class I–II	20 (16)	7 (19)	
Class II	33 (27)	14 (38)	
Class II–III	6 (5)	2 (5)	
Class III	6 (5)	2 (5)	
Duration of heart failure			0.419
<1 year	6 (5)	1 (3)	
1–5 years	57 (47)	24 (65)	
≥ 5 years	57 (47)	12 (32)	

Numbers are *n* (%) or mean ± standard deviation

*TNA* nonfocal transient neurological attack, *Q1–Q3* interquartile range, *COPD* chronic obstructive pulmonary disease, *OSAS* obstructive sleep apnea syndrome, *TIA* transient ischemic attack, *PAOD* peripheral arterial occlusive disease, *LVEF* left ventricular ejection fraction, *NYHA* New York Heart Association

Table 3 shows the *z* scores of each cognitive domain for patients with and without TNAs. Patients with TNAs performed significantly worse than patients without TNAs on global cognition, language, memory and attention-psychomotor speed, but not on executive functioning. Impairment of ≥ 1 cognitive domains was more frequent in patients with than in patients without TNAs [41% vs. 18%; adjusted odds ratio (OR) 4.6, 95% confidence interval (CI) 1.8–11.8]. Impairment of ≥ 2 cognitive domains was also more frequent in patients with TNAs than in patients without TNAs (19% vs. 4%; adjusted OR 10.4, 95% CI 2.4–45.5) (Table 4). Additional adjustments for volume of white matte hyperintensities (mL) (Tables 3, 4) and for cardiac output (Supplemental Tables 1 and 2) did not influence these results.

**Table 3:** *z* scores per cognitive domain and results from the linear regression analysis of the association of TNA with *z* scores of cognitive functioning

	No TNA (*n* = 121)	≥ 1 TNA (*n* = 37)	Mean difference (95% CI)^a^	*P* value	Mean difference (95% CI)^b^	*P* value
	Mean ± SD *z* score	Mean ± SD *z* score				
Global cognition	− 0.30 ± 0.6	− 0.64 ± 0.6	− 0.36 (− 0.54 to − 0.18)	0.000	− 0.39 (− 0.57 to − 0.20)	0.000
Cognitive domain						
Attention-psychomotor speed	− 0.39 ± 0.8	− 0.79 ± 0.9	− 0.40 (− 0.66 to − 0.14)	0.003	− 0.44 (− 0.72 to − 0.16)	0.002
Language	− 0.27 ± 0.7	− 0.75 ± 1.3	− 0.47 (− 0.79 to − 0.16)	0.003	− 0.30 (− 0.58 to − 0.01)	0.041
Memory	− 0.33 ± 1.2	− 0.80 ± 1.3	− 0.57 (− 0.98 to − 0.15)	0.008	− 0.73 (-1.15 to − 0.31)	0.001
Executive functioning	− 0.22 ± 0.8	− 0.22 ± 0.8	− 0.01 (− 0.27 to 0.26)	0.953	− 0.08 (− 0.36 to 0.21)	0.594

*TNA* nonfocal transient neurological attack, *SD* standard deviation, *mean difference*, the mean difference in *z* score of cognitive performance for ≥ 1 vs. no TNA, *CI* confidence interval

^a^Adjusted for age, sex and education

^b^Adjusted for age, sex, education and white matter hyperintensities volume (mL)

**Table 4:** Odds ratios for cognitive impairment within 6 months after TNA compared with patients without TNA

	No TNA (*n* = 121)	≥ 1 TNA (*n* = 37)	Cognitive impairment, OR (95% CI) adjusted^a^	*P* value	Cognitive impairment, OR (95% CI) adjusted^b^	*P* value
≥ 1 Cognitive domains	22 (18)	15 (41)	4.6 (1.8–11.8)	0.002	5.3 (1.9–14.4)	0.001
≥ 2 Cognitive domains	5 (4)	7 (19)	10.4 (2.4–45.5)	0.002	14.1 (2.8–71.9)	0.001

Numbers are *n* (%) unless stated otherwise

Cognitive impairment defined as domain *z* score < − 1.5

*OR* odds ratio, *CI* confidence interval, *TNA* nonfocal transient neurological attack

^a^Adjusted for age, sex and education

^b^Adjusted for age, sex, education and white matter hyperintensities volume (mL)

Twenty-three patients (15%) had a previous stroke or TIA, and 73 patients (46%) had visible brain infarction on MRI. Subgroup analyses in patients without brain infarction on MRI or previous stroke or TIA (Tables 5, 6, Supplemental Tables 3 and 4) yielded comparable results, with the exception of the cognitive domain language, in which there was no longer a difference between patients with and without TNAs.

**Table 5:** Sensitivity analysis in patients without previous stroke or TIA and without brain infarction on MRI (*n* = 78)

	No TNA (*n* = 62)	≥ 1 TNA (*n* = 16)	Mean difference (95% CI)^a^	*P* value	Mean difference (95% CI)^b^	*P* value
	Mean ± SD *z* score	Mean ± SD *z* score				
Global cognition	− 0.14 ± 0.6	− 0.62 ± 0.7	− 0.36 (− 0.59 to − 0.12)	0.004	− 0.35 (− 0.59 to − 0.12)	0.004
Cognitive domain						
Attention-psychomotor speed	− 0.21 ± 0.7	− 0.92 ± 1.1	− 0.51 (− 0.87 to − 0.15)	0.006	− 0.51 (− 0.87 to − 0.15)	0.006
Language	− 0.27 ± 0.8	− 0.45 ± 0.4	− 0.04 (− 0.45 to 0.38)	0.865	− 0.33 (− 0.45 to 0.38)	0.876
Memory	− 0.09 ± 0.9	− 0.84 ± 1.5	− 0.68 (− 1.25 to − 0.11)	0.020	− 0.68 (− 1.25 to − 0.11)	0.021
Executive functioning	− 0.01 ± 0.6	− 0.27 ± 0.8	− 0.20 (− 0.53 to 0.13)	0.238	− 0.20 (− 0.53 to 0.14)	0.243

*z* scores per cognitive domain and results from the linear regression analysis of the association of TNA with *z* scores of cognitive functioning

*TNA* nonfocal transient neurological attack, *SD* standard deviation, *mean difference* the mean difference in *z* score of cognitive performance for ≥ 1 vs. no TNA, *CI* confidence interval

^a^Adjusted for age, sex and education

^b^Adjusted for age, sex, education and white matter hyperintensities volume (mL)

**Table 6:** Sensitivity analysis in patients without previous stroke or TIA and without brain infarction on MRI (*n* = 78)

	No TNA (*n* = 62)	≥ 1 TNA (*n* = 16)	Cognitive impairment, OR (95% CI) adjusted^a^	*P* value	Cognitive impairment, OR (95% CI) adjusted^b^	*P* value
≥ 1 Cognitive domains	6 (10)	6 (38)	5.8 (1.1–29.9)	0.035	5.8 (1.1–29.7)	0.036
≥ 2 Cognitive domains	1 (2)	3 (19)	16.6 (1.1–253.8)	0.044	19.3 (1.1–354.5)	0.046

Odds ratios for cognitive impairment within 6 months after TNA compared with patients without TNA

Numbers are *n* (%) unless stated otherwise

Cognitive impairment defined as domain *z* score < − 1.5

*OR* odds ratio, *CI* confidence interval, *TNA* nonfocal transient neurological attack

^a^Adjusted for age, sex and education

^b^Adjusted for age, sex, education and white matter hyperintensities volume (mL)

## Discussion

Patients with heart failure who had a recent TNA had a higher risk of impairment in ≥ 1 and ≥ 2 cognitive domains than similar patients without TNAs. This difference concerned global cognition, as well as the cognitive domains language, memory and attention-psychomotor speed. The results were not influenced by cardiac output or volume of white matter hyperintensities. Subgroup analyses in patients without brain infarction on MRI or previous stroke or TIA yielded comparable results, with the exception of the cognitive domain language.

To the best of our knowledge, this is the first study addressing cognitive functioning after a TNA. Subjective cognitive complaints, as measured by the Cognitive Failures Questionnaire, have been investigated in patients who had a TIA or TNA [25]. Six months after the initial event, 77% of these patients experienced subjective cognitive complaints [25]. The authors did not provide separate results after TNA only, but they stated that subjective cognitive complaints were the same in patients diagnosed with a TNA as in patients with a TIA [25]. Another study investigated the 6-month course of cognitive functioning after both TIA and TNA combined, in relation to diffusion weighted imaging (DWI) results at baseline [26]. Patients with a DWI lesion had worse executive function at baseline than those without a DWI lesion, which persisted throughout the 6-month study period [26]. The authors mentioned that the clinical diagnosis (TIA or TNA) was not related to cognitive functioning at baseline or over time, even though numbers were not provided separately for patients with a TNA only [26]. Although the above-described studies have a different design, the results seem to concur with our findings [25, 26].

There are several mechanisms that may describe the relation between a recent TNA and cognitive impairment. First, as TNAs and cognitive impairment share some of the same risk factors, they may result from the same underlying disease process [1, 27, 28]. Nevertheless, in our study sample, patients with TNAs were not more likely to have vascular risk factors, such as hypertension, lipid-lowering medication or diabetes mellitus than participants without TNAs. Second, cognitive impairment could be the result of cerebral hypoperfusion, which has long been suggested a potential cause of TNAs [1, 2, 29]. This concurs with the results of a large, population-based study in which cerebral hypoperfusion is associated with accelerated cognitive decline [30]. Previous studies in heart failure patients that related the left ventricular ejection fraction to cognitive impairment showed conflicting results [9, 10, 31]. However, in our study, cardiac output, left ventricular ejection fraction and NYHA classification did not differ between patients with and without TNAs, and adjusting for cardiac output did not influence our results. Cardiac output may serve as a surrogate for the severity of heart failure, and consequently the extent to which cerebral perfusion potentially may be compromised [6–8, 32, 33]. Therefore, our findings argue against the explanation that cognitive impairment in patients with TNAs is mediated through cerebral hypoperfusion [6–8, 32, 33].

A third explanation is that, even though TNAs are by definition transient, they might lead to permanent brain damage. This brain damage could disrupt networks leading to cerebral atrophy and cognitive impairment [34]. This explanation is compatible with the results of previous studies in which similar signs of acute focal cerebral ischemia, as measured by diffusion weighted imaging, were found in patients diagnosed with both a recent TIA and recent TNA [4, 35]. Our finding that patients with TNAs more often had a history of TIA than patients without TNAs supports this hypothesis. Still, our results were not substantially influenced after excluding all patients with previous stroke or TIA or brain infarction on MRI, which might argue against permanent brain injury as the cause of cognitive impairment in patients with TNAs. Furthermore, adjusting for volume of white matter hyperintensities on MRI did not change our results. In conclusion, the exact mechanisms between TNAs and cognitive impairment remain unclear.

Our second finding that patients with TNAs perform significantly worse than patients without TNAs on global cognition, language, memory and attention-psychomotor speed, but not on executive functioning is remarkable, as we had expected that executive functioning would be the most sensitive cognitive domain [26]. There are several explanations for this finding. A first possibility is that our neurocognitive tests were not sensitive enough to detect changes in executive functioning. However, we used neuropsychological tests with international standards (TMT B/A index, SCWT III, SCWT interference and Digit span backward) to measure executive functioning. Second, we intentionally separated executive functioning from attention/psychomotor speed, while some authors chose to combine both domains into one cognitive domain [36].

Certain limitations of our study have to be considered. First, as this is a cross-sectional study, we cannot draw conclusions on causality. Second, the diagnosis of a TNA is made only on the basis of self-report and we may either have misclassified episodes as TNAs or missed TNAs that were not remembered by the patient, leading to recall bias. However, we used a standardized questionnaire, systematically assessed by a trained physician or research nurse, which covered all types of TNAs we were interested in. Third, as we studied patients with heart failure, the generalizability to a broader group of patients with TNAs is limited. However, our patient population was chosen deliberately, as patients with heart failure may be vulnerable for cerebral hypoperfusion through a compromised cardiac output. Most patients with heart failure had NYHA class I or I–II, indicating they had only mild heart failure, which might implicate that our results are not generalizable to patients with severe heart failure. Last, we have no information regarding the functional impact of cognitive problems on the daily living in patients with TNAs or on subjective memory complaints before and after the TNA.

One of the strengths of our study is that we conducted a comprehensive neuropsychological test battery composed of tests with international standards, administered by trained neuropsychologists. Therefore, we were able to draw conclusions on domain-specific cognitive functioning. A novelty of this study is that we focused on patients diagnosed with TNAs only, instead of patients diagnosed with TNAs and TIAs combined. Of note, a TNA is not a specific clinical diagnosis, but rather an entity that includes many different symptoms. It is a concept that is used to describe those patients that are clinically not diagnosed as having had a TIA [3]. Although they are traditionally seen as a benign event, they are associated with an increased risk of future vascular events and dementia [2, 4, 5]. Our results further emphasize the need to change this widely accepted benign concept of TNAs into a more hazardous event.

In conclusion, among patients with heart failure, TNAs are related to cognitive impairment. This highlights the need for more clinical awareness of both TNAs and cognitive impairment in heart failure patients. Future studies are needed to unravel the mechanisms leading to the association between cognitive impairment and TNAs, and to determine the effect of therapeutic intervention. These studies should be longitudinal and include advanced brain imaging techniques to identify ischemic, microstructural and perfusion changes.

## Electronic supplementary material

Below is the link to the electronic supplementary material.

Supplementary material 1 (PDF 148 kb)
