# Supplementary material for: Nonfocal transient neurological attacks are related to cognitive impairment in patients with heart failure
Source: J Neurol. 2019 May 21;266(8):2035–42. doi: 10.1007/s00415-019-09376-z (PMC6647193; doi:10.1007/s00415-019-09376-z)
Supplement: Supplementary file 1 — Supplementary material 1 (PDF 148 kb) [file 415_2019_9376_MOESM1_ESM.pdf]

SUPPLEMENTAL MATERIAL

Journal of Neurology

Nonfocal transient neurological attacks are related to cognitive impairment in patients with heart failure.

Eline A. Oudeman MD[1,2], Jacoba P. Greving PhD[3], Astrid M. Hooghiemstra PhD[4,5], H.P. Brunner-La Rocca[6], Geert Jan Biessels[1], L. Jaap Kappelle MD PhD[1], on behalf of the HBC- Study Group

1. Department of Neurology and Neurosurgery, Brain Centre Rudolf Magnus, University Medical Centre Utrecht, Utrecht University, Utrecht, the Netherlands.
2. Department of Neurology, OLVG west, Amsterdam, the Netherlands.
3. Julius Centre for Health Sciences and Primary Care, University Medical Centre Utrecht, Utrecht University, Utrecht, the Netherlands
4. Alzheimer Center Amsterdam, Department of Neurology, Amsterdam Neuroscience, Vrije Universiteit Amsterdam, Amsterdam UMC, Amsterdam, The Netherlands.
5. Department of Medical Humanities, Amsterdam Public Health Research Institute, Amsterdam UMC, Vrije Universiteit Amsterdam, Amsterdam, The Netherlands
6. Department of Cardiology, Maastricht University Medical Center, Maastricht, the Netherlands.

Email of the corresponding author: e.a.oudeman@gmail.com

Online Resource 1. Z-scores per cognitive domain and results from the linear regression analysis of the association of TNA with z-scores of cognitive functioning with additional adjustments for cardiac output.

	**No TNA (n= 121)**	**≥1 TNA (n= 37)**	**Mean difference (95% CI) [a]**	**P-value**	**Mean difference (95% CI) [b]**	**P-value**
	**Mean ± SD z-score**	**Mean ± SD z-score**				
Global cognition	-.30 ± 0.6	-.64 ± 0.6	-.36 (-.54 to -.18)	0.000	-.34 (-.54 to -.15)	0.001
Cognitive domain						
Attention- psychomotor speed	-.39 ± 0.8	-.79 ± 0.9	-.40 (-.66 to -.14)	0.003	-.40 (-.67 to -.12)	0.005
Language	-.27 ± 0.7	-.75 ± 1.3	-.47 (-.79 to -.16)	0.003	-.37 (-.68 to -.05)	0.022
Memory	-.33 ± 1.2	-.80 ± 1.3	-.57 (-.98 to -.15)	0.008	-.56 (-.99 to -.12)	0.013
Executive functioning	-.22 ± 0.8	-.22 ± 0.8	-.01 (-.27 to .26)	0.953	-.05 (-.33 to .23)	0.718

TNA indicates nonfocal transient neurological attack; SD, standard deviation; mean difference, the mean difference in z-score of cognitive performance for ≥1 vs. no TNA; CI, confidence interval.

[a] Adjusted for age, sex and education.

[b] Adjusted for age, sex, education and cardiac output.

Online Resource 2. Odds ratios for cognitive impairment within 6 months after TNA compared with patients without TNA with additional adjustments for cardiac output.

	No TNA (n=121)	≥1 TNA (n= 37)	Cognitive impairment, OR (95%- CI) Adjusted [a]	P-value	Cognitive impairment, OR (95%- CI) Adjusted [b]	P-value
≥1 cognitive domains	22 (18)	15 (41)	4.6 (1.8 - 11.8)	0.002	3.4 (1.3 – 9.1)	0.014
≥2 cognitive domains	5 (4)	7 (19)	10.4 (2.4 - 45.5)	0.002	9.8 (2.1 – 46.9)	0.004

Numbers are n (%) unless stated otherwise.

Cognitive impairment defined as domain z score < -1.5.

OR indicates odds ratio; CI, confidence interval; TNA, nonfocal transient neurological attack.

[a] Adjusted for age, sex and education.

[b] Adjusted for age, sex, education and cardiac output.

Online Resource 3. Sensitivity analysis in patients without previous stroke or TIA and without brain infarction on MRI (n=78). Z-scores per cognitive domain and results from the linear regression analysis of the association of TNA with z-scores of cognitive functioning with additional adjustments for cardiac output.

	**No TNA (n= 62)**	**≥1 TNA (n= 16)**	**Mean difference (95% CI) [a]**	**P-value**	**Mean difference (95% CI) [b]**	**P-value**
	**Mean ± SD z-score**	**Mean ± SD z-score**				
Global cognition	-.14 ± 0.6	-.62 ± 0.7	-.36 (-.59 to -.12)	0.004	-.37 (-.61 to -.13)	0.003
Cognitive domain						
Attention- psychomotor speed	-.21 ± 0.7	-.92 ± 1.1	-.51 (-.87 to -.15)	0.006	-.51 (-.89 to -.14)	0.008
Language	-.27 ± 0.8	-.45 ± 0.4	-.04 (-.45 to .38)	0.865	-.01 (-.42 to .44)	0.975
Memory	-.09 ± 0.9	-.84 ± 1.5	-.68 (-1.25 to -.11)	0.020	-.76 (-1.34 to -.18)	0.012
Executive functioning	-.01 ± 0.6	-.27 ± 0.8	-.20 (-.53 to .13)	0.238	-.21 (-.55 to .14)	0.234

TNA indicates nonfocal transient neurological attack; SD, standard deviation; mean difference, the mean difference in z-score of cognitive performance for ≥1 vs. no TNA; CI, confidence interval.

[a] Adjusted for age, sex and education.

[b] Adjusted for age, sex, education and cardiac output.

Online Resource 4. Odds ratios for cognitive impairment within 6 months after TNA compared with patients without TNA with additional adjustments for cardiac output.

	No TNA (n=62)	≥1 TNA (n= 16)	Cognitive impairment, OR (95%- CI) Adjusted [a]	P-value	Cognitive impairment, OR (95%- CI) Adjusted [b]	P-value
≥1 cognitive domains	6 (10)	6 (38)	5.8 (1.1 – 29.9)	0.035	5.9 (1.1 – 32.5)	0.041
≥2 cognitive domains	1 (2)	3 (19)	16.6 (1.1 - 253.8)	0.044	16.0 (1.0 – 247.4)	0.047

Numbers are n (%) unless stated otherwise.

Cognitive impairment defined as domain z score < -1.5.

OR indicates odds ratio; CI, confidence interval; TNA, nonfocal transient neurological attack.

[a] Adjusted for age, sex and education.

[b] Adjusted for age, sex, education and cardiac output.
